# Review Article: Accelerating Coeliac Disease Clinical Trials—Beyond Celiac Meeting With Industry, Academics, Patients and the FDA

**DOI:** 10.1111/apt.70873

**Published:** 2026-07-28

**Authors:** Lisa M. Fahey, Robert Anderson, Edwin Liu, Joseph A. Murray, Marie E. Robert, Jocelyn Anne Silvester, Kate Avery, Nelson Cheinquer, Kristie Grebe, Bhargav Tulabandula, Daniel A. Leffler, Beyond Celiac Coalition

**Affiliations:** ^1^ Children's Hospital of Philadelphia Philadelphia Pennsylvania USA; ^2^ University of Pennsylvania Philadelphia Pennsylvania USA; ^3^ International Society for the Study of Celiac Disease Groningen the Netherlands; ^4^ Mackay Base Hospital Mackay Queensland Australia; ^5^ Children's Hospital Colorado University of Colorado School of Medicine Aurora Colorado USA; ^6^ Mayo Clinic College of Medicine and Science Rochester Minnesota USA; ^7^ Yale University School of Medicine New Haven Connecticut USA; ^8^ Boston Children's Hospital Boston Massachusetts USA; ^9^ Harvard Medical School Boston Massachusetts USA; ^10^ Beyond Celiac Ambler Pennsylvania USA; ^11^ Takeda Cambridge Massachusetts USA; ^12^ Anokion Cambridge Massachusetts USA; ^13^ Navigator Medicines Cambridge Massachusetts USA; ^14^ Chugai Pharmaceuticals Berkeley Height New Jersey USA

**Keywords:** Beyond Celiac Coalition, coeliac disease, drug development, Food and Drug Administration

## Abstract

**Background:**

Coeliac disease is due to an acquired, lifelong gluten‐specific immune response resulting in a gluten‐dependent chronic immune‐mediated enteropathy associated with gastrointestinal and extraintestinal morbidity. Strictly avoiding dietary gluten is the only available management but has never been subjected to rigorous efficacy assessment now required for a pharmaceutical agent. Gluten‐free diet (GFD) is partially and inconsistently effective, but it places a high burden on patients, families, the food industry, and regulators and is defined inconsistently around the world, highlighting the need for effective and convenient adjunctive pharmacological therapies.

**Aims:**

To summarize key patient, scientific, and regulatory considerations for coeliac disease drug development based on a multi‐stakeholder meeting focused on accelerating clinical trials.

**Methods:**

A pre‐competitive meeting convened by the Beyond Celiac Coalition (May 2024) included patients with coeliac disease, academic experts, pharmaceutical industry representatives, and participants from the US Food and Drug Administration. Moderated panel addressed patient priorities, symptom and histologic endpoints, biopsy and histology methods, gluten challenge design, clinical trial duration, and regulatory expectations. Themes were synthesized qualitatively with focus on trial design implications and unresolved questions.

**Results:**

Participants described high symptom burden and psychosocial impact despite gluten‐free diet adherence underscoring unmet therapeutic need in coeliac disease. Regulatory perspectives emphasized the need for clinically meaningful improvement and evidence of intestinal benefit, framed as symptom and histology co‐primary endpoints in clinical trials. These expectations were discussed as most applicable to trial populations with both clinically meaningful symptoms and active histologic injury at baseline; for other phenotypes, it was emphasized that endpoint strategy, the role of gluten exposure, and trial duration should be tailored case by case to the population and mechanism of action. Practical challenges identified included endpoint selection and definition, discordance between symptoms and histology, selecting fit‐for‐purpose symptom measures; standardizing biopsy acquisition and central histology reads to address patchiness and variability, pre‐specifying definitions of meaningful histologic change given ongoing uncertainty about healing, and trial feasibility. Controlled gluten exposure was discussed as a tool to improve interpretability of efficacy outcomes, with careful consideration of dose, duration, patient burden and safety. Discussion addressed the ideal length of the double‐blind period, including a shorter 24‐week double‐blind study with an open‐label extension versus a 52‐week double‐blind design. Trial duration should balance scientific rigour, patient retention, and the need for longer‐term safety and durability data, with flexibility to tailor the approach to context.

**Conclusions:**

Progress in coeliac disease drug development will require patient‐centred, scientifically robust but feasible trial designs with appropriate endpoints, justified use of gluten exposure, consideration of improved biomarkers to reduce patient burden and early regulatory engagement. Continued multi‐stakeholder collaboration, including dedicated consideration of paediatric drug development, is essential to advance effective therapies.

## Graphical abstract

A 2024 multi‐stakeholder meeting examined how to accelerate coeliac disease drug trials, highlighting key considerations in endpoint selection, symptom–histology alignment and feasibility. Progress requires patient‐centred trials showing symptomatic and intestinal improvement, appropriate gluten challenge, and early regulatory engagement, including paediatric development.

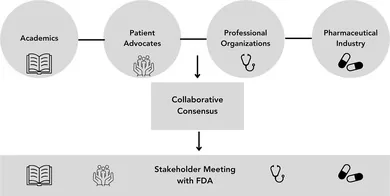

## 1 Introduction

Coeliac disease is a chronic autoimmune disorder caused by an acquired, lifelong gluten‐specific immune response resulting in gluten‐dependent enteropathy and acute and chronic gluten‐dependent non‐GI consequences. Untreated coeliac disease commonly produces diarrhoea, weight loss, abdominal pain, malnutrition, iron deficiency and other mineral deficiencies. Patients with coeliac disease normally excluding gluten can also experience transient dose‐dependent acute upper‐gastrointestinal reactions after ingestion of gluten that closely linked to immune activation with systemic cytokine release [1]. Patients with coeliac have a higher risk of many diverse life and health impacting consequences including bone disease (e.g., osteoporosis, osteomalacia and fractures), female and male infertility, malignancies including T‐cell lymphoma or small bowel cancer, and cardiovascular disease despite lower overall risk profile for cardiovascular disease. The burden for patients of adhering to a life‐long gluten free diet as the only management for coeliac disease is similar to diabetes and greater than inflammatory bowel disease and irritable bowel syndrome [2]. Coeliac disease and its current reliance on a strict self‐managed exclusion diet have significant impact on quality of life [3].

A century has passed since Dr. Sidney Haas pioneered the banana diet to treat coeliac disease and 70 years since Dicke identified gluten as the driver of coeliac disease. As we enter 2026, it is hard to believe that coeliac patients world‐wide still do not have alternatives to the gluten‐free diet despite the obvious burden as well as inconsistent efficacy of the gluten‐free diet. There is, however, a detailed understanding of coeliac disease pathogenesis, well‐defined traditional and emerging biomarkers and therapeutic targets, and many investigational products are advancing through clinical trials stimulating constructive dialogue to efficiently navigate the regulatory hurdles necessary for the first therapeutic agents to be approved for coeliac disease. It is therefore critical that academic researchers, clinical experts, biopharmaceutical industry partners, patient advocacy groups and regulators are well informed to allow effective collaboration leading to approval of pharmaceuticals for patients inadequately managed by gluten‐free alone.

Prompted by the FDA 2022 draft guidance on drug development for coeliac disease [4], the Beyond Celiac Coalition, a pre‐competitive partnership of multidisciplinary members involved in the development of potential treatments for coeliac disease, organized an agenda and conducted a meeting with representatives from the FDA in Bethesda, Maryland in May 2024 with other stakeholders that included patients, industry representatives and academic experts in coeliac disease.

The goal was to bring together patients, industry and clinician–scientist experts to discuss key considerations for scientifically rigorous, patient‐centred coeliac disease trials, informed by recent academic perspectives, and to discuss the draft FDA guidance [4] and seek clarification from the FDA in order to advance a collaborative approach to designing scientifically sound patient‐centric therapeutic trials for coeliac disease.

## 2 Methods

### 2.1 Participants

The Beyond Celiac Coalition is comprised of industry, academic and professional organization members. Industry members pay a membership fee to join the Coalition. Academicians are individually invited to join the Coalition based on their relevant interest in coeliac disease. Many of the academic members are part of coeliac centres across the United States. Professional organizations are invited to join based on mutual interest.

For this meeting, the Coalition invited adult patients and FDA representatives to participate. Patient representatives were chosen by Beyond Celiac. All were symptomatic with gluten exposure. Patient representatives were of various ages, time to diagnosis, and symptoms with gluten exposure. FDA representatives were invited through the official Speaker Request process. The individual centres decided which FDA employees participated in the workshop.

The Beyond Celiac Coalition reimbursed academicians and patients for travel and lodging expenses. There was no other compensation for participants. Industry partners paid a fee to Beyond Celiac to be a part of the Beyond Celiac Coalition, as well as to attend this meeting. The FDA did not pay or accept payment for their participation in this meeting.

Meeting notes were taken by several meeting participants, including Beyond Celiac Coalition members. The notes were synthesized by the manuscript authors who are also members of the Beyond Celiac Coalition.

### 2.2 Agenda Creation

The overall meeting agenda and talking points were decided among Coalition members during a series of discussions in the months leading up to the workshop. All Coalition members had the opportunity to provide comments and shape the agenda.

## 3 Results

### 3.1 Meeting Key Concepts

#### 3.1.1 Topic 1: Coeliac Disease Patient Perspectives

Panellists frequently described delayed, missed, or chance‐driven diagnosis, including years of symptoms attributed to other causes (e.g., irritable bowel syndrome) or minimized by others, with diagnosis sometimes occurring only when a clinician with personal familiarity suggested testing. After diagnosis, symptoms related to gluten exposure can vary widely and can be debilitating for some. Beyond physical symptoms, the panellists emphasized the significant psychosocial burden associated with following a gluten free diet that can be socially isolating and can negatively impact mental health [5]; captured by one participant comment that ‘the spontaneity in my life has been wiped out’. Fear of eating and decreased school or job performance were highlighted as two examples of this negative impact. Coeliac disease was repeatedly framed as an ‘invisible illness’, which can lead others to minimize their condition or think they might ‘get over it’ with time. They also described anxiety about gluten exposure, especially with eating out or when food is out of their control, with one panellist describing not eating before presentations for work because ‘you can't risk it’. Looking ahead, patient panellists called for easier, earlier diagnosis and progress through clinical research and clinical trials toward therapies that could enable them to ‘lead normal lives’.

#### 3.1.2 Topic 2A: Signs and Symptoms of Coeliac Disease

Coeliac disease symptoms are heterogeneous and it is challenging for both patients and clinicians to determine if these symptoms reflect acute, intermittent or chronic gluten exposure, persistent end‐organ intestinal injury or an alternative cause unrelated to coeliac disease [6]. Medical misdiagnosis may further complicate evaluation where one case series reported 20% medical misdiagnosis [7]. Persistent symptoms may therefore reflect continued gluten exposure, incomplete response to a gluten‐free diet, or an alternative diagnosis or co‐morbidity.

Against this clinical backdrop, the stakeholder discussion considered coeliac drug development needs in the context of the draft FDA guidance [4], which recognizes the unmet need for therapies intended as adjuncts to, rather than replacements for dietary management. Panellists noted that many patients remain symptomatic and/or have persistent intestinal injury despite best efforts at dietary adherence, commonly described in clinical practice under the umbrella of non‐responsive coeliac disease. This heterogeneity underscored a central theme of the meeting: trial interpretability depends on clearly defining the target patient phenotype and inclusion criteria, because these choices shape endpoint selection/definition, feasibility and the role of gluten exposure.

#### 3.1.3 Topic 2B: Endpoints, Histology and Feasibility

Discussion by all parties was interpreted in the context of the draft FDA guidelines acknowledging unmet need for pharmaceuticals as adjuncts but not replacement for dietary therapy. Measurement of both symptoms and histologic findings is reflective of active coeliac disease ‘unresolved’ or ‘partially’ improved by dietary management in many patients and referred to as ‘non‐responsive coeliac disease’ [8, 9, 10]. Indeed, many trials now conducted and frequently cited academic reviews advocate enrollment of patients with normal/near‐normal duodenal histology to show whether an investigational product can mitigate intestinal injury and symptoms associated with calibrated gluten challenge over a period of weeks or months [11, 12]. Consistent with its guidelines for drug developers in several other gastrointestinal diseases, the FDA participants noted that the recommendation to evaluate efficacy by both coeliac disease‐relevant patient‐reported outcomes, as well as objective measurement of intestinal injury by histology as co‐primary endpoints.

FDA representatives noted that trial duration should be sufficient to capture expected histological improvement and that, as current programs use varied symptoms and histologic endpoints, further data will be important to support consensus on clinically meaningful, standardized endpoint definitions for regulatory decision‐making. Importantly, FDA representatives emphasized that the draft guidance [4] is intended as a framework to advance the field and that some patient subgroups are not directly addressed by the recommended trial design approach, including asymptomatic patients.

The FDA representatives highlighted that if a drug demonstrates improvement on both co‐primary endpoints (i.e., symptoms and histology) in a symptomatic population enrolled in the clinical trials and receives approval as a treatment for coeliac disease, the information on the histologic co‐primary endpoint would be relevant for treating asymptomatic patients who have active histologic findings, or patients with ongoing symptoms without significant intestinal injury, provided there are no concerns with the efficacy or safety data that would restrict use to a specific population.

The Coalition highlighted practical challenges regarding the design of a phase 3 trial that could achieve both co‐primary endpoints of symptom and histologic improvement for coeliac clinical trials with or without reintroduction of gluten (see below). Coalition members expressed concern that co‐primary endpoints within one trial will be difficult to achieve because of a lack of correlation between histology and symptoms in coeliac patients. There was discussion of whether phase 3 trials could support drug approval if investigational products were associated with either improvement of duodenal histology or symptom improvement and so potentially allow two distinct study designs. Some members of the Coalition advocated that achieving one of the endpoints, rather than both, should be sufficient for a successful trial. From the patient perspective, either improvement in histology (even if not completely healed) or improvement in symptoms without worsening of histology would provide evidence of improved disease control.

FDA representatives clarified that drugs intended for the treatment of coeliac disease should improve symptoms, as well as treat the underlying histopathology. While reducing pain or discomfort is critical, it is equally important to reduce intestinal damage caused by gluten as the histologic damage directly reflects the underlying disease process. The co‐primary endpoint approach is typically utilized when there is a lack of correlation between symptoms and histology, which is common in patients with coeliac disease. The co‐primary endpoint approach also allows for demonstrating effectiveness in a clinical trial by achieving each endpoint at the population level, rather than achieving both at the individual patient level. The clinical trials should be of adequate duration to evaluate the amount of improvement in histology that would be expected within the timeframe of the study. Currently, individual trials measure a variety of different endpoints; however, as more data are collected, the goal is to gain consensus on the relevant clinical and histological endpoint definitions that could support approval of a drug.

Standardization of histology was identified as a key need and a major determinant of trial feasibility. There was agreement that confirming coeliac disease is essential and that histology remains the most established objective marker of disease activity, while also acknowledging limitations of biopsy‐based assessment, including sampling constraints and patchiness of mucosal injury. Participants highlighted gaps in defining and operationalizing duodenal healing, including the possibility that intraepithelial lymphocyte normalization may lag behind villous architectural recovery [13]. Consistent approaches to biopsy acquisition, processing and central reading were therefore viewed as important to improve interpretability and comparability across trials.

Important limitations remain in assessing global coeliac disease activity using biopsies from the distal duodenum alone. Intestinal injury results from gluten (antigen) exposure and the ‘strength’ of an individual's immune response to gluten, which is known to vary substantially based on gluten bolus‐stimulated cytokine release and acute symptoms, and the frequency of circulating gluten‐reactive T cells before and after gluten challenge [14, 15, 16].

#### 3.1.4 Topic 2C: Immune Activity and Biomarkers as Potential Complements to Histology in the Future

Participants discussed biological variability in gluten‐driven immune activation, including differences in cytokine responses and the frequency of circulating gluten‐reactive T cells before and after gluten exposure [14, 15, 16]. While serology is widely used clinically to support diagnosis and to monitor response to a gluten‐free diet, it was described as exploratory for use as a clinical trial endpoint given current limitation in evidence linking serologic changes to changes in disease activity, progression, and treatment response over time. Ongoing research is rapidly expanding understanding of serum cytokines and ex vivo cytokine release assays (e.g., interleukin‐2 associated with gluten stimulation), which may offer practical alternatives in the future to endpoints requiring extended gluten challenge or histology especially in paediatric studies [17].

### 3.2 Topic 3: Using Gluten as a Drug Development Tool

Many current drugs under development are designed to protect patients from the effects of gluten exposure. As real‐world gluten exposure cannot be accurately measured or controlled, including carefully controlled and monitored gluten exposure in coeliac disease studies can provide more confidence in drug efficacy. In a clinical trial context, gluten is used to evoke the immune response causing the symptoms and intestinal injury that a drug is intended to mitigate or prevent. The Coalition highlighted that while chronic (i.e., years) gluten exposure is problematic, symptoms and intestinal damage induced by short term (days to months) gluten challenges during clinical trials will resolve when a gluten free diet is resumed [18]. There was discussion about the best way to include gluten in trials because some trial participants drop out quickly due to symptoms when gluten is reintroduced at high doses. With sustained daily gluten exposure, symptoms may change or decrease. From a patient perspective, brief, higher dose gluten challenges and extended lower dose (intermittent) gluten exposures may have different impacts that can affect trial participation. There also may be geographical or cultural differences in the amount of gluten that an individual is willing to consume during a trial [19].

Ultimately, the amount of gluten needed is contingent upon whether the study design is intended to assess symptoms or to assess histologic recovery. Symptom assessment may involve intermittent, moderate (e.g., > 2 g) doses of gluten. Histologic recovery assessment may not include gluten exposure or involve regular, small doses (e.g., 0.5 mg). Additionally, while not validated for use as a clinical trial endpoint, immunologic endpoints yield more immediate readouts (e.g., change in serum interleukin‐2 at 2–6 h after bolus gluten) correlated with severity and timing of acute digestive symptoms than histologic endpoints [14, 20, 21]. The importance of composition of gluten challenges both in terms of patient acceptability and scientific interpretability were also highlighted.

Finally, FDA representatives emphasized that the amount and duration of gluten administered should be justified and aligned with the trial objectives, mechanism of action of the drug, and the scientific question the study design is intended to address. Across discussions, there was consensus that patient education and patient monitoring are important for both the success and safety of coeliac disease drug development.

### 3.3 Topic 4: Clinical Trial Length

The final panel discussion highlighted that patient panellists would generally be willing to participate in a clinical trial even if they may get placebo. Regarding trial length, the Coalition discussed that a 1‐year clinical trial duration is challenging from both patient burden and patient retention standpoints. This is uniquely challenging for coeliac disease because the gluten‐free diet already imparts so much burden that the added clinical trial burden can sometimes be too much. There was discussion by the Coalition that a 24‐week controlled study with open‐label extension to 52 weeks should be strongly considered as it provides a balance of scientific rigour and patient burden, while providing important data on outcomes when patients know they are on active therapy, which may lead to changes in gluten exposure. There was also discussion that all drugs are currently being designed as an adjunct to, rather than a replacement for, the gluten‐free diet, and that this should be explained to trial participants.

FDA representatives noted that coeliac disease is a chronic condition and there is a need for safety and efficacy data of adequate duration to inform ongoing administration of a drug over years of a patient's lifetime. They recommended assessing durability of response over 52 weeks in a controlled trial; however, the assessment of primary endpoints could occur at an earlier timepoint (e.g., 24 weeks). While there is agreement that trials should be designed keeping patient burden in mind, it is important to obtain the most interpretable data that would support drug approval, which may be limited by potential bias introduced with an open‐label study design.

## 4 Conclusions

There were important clarifications that arose from the meeting (Table 1):

1. To support efficacy, the FDA representatives maintained that a successful trial must achieve statistical significance for both primary endpoints, patient reported outcomes and quantitative histology to support a therapeutic label claim in coeliac disease. For others in the Coalition, there was continuing scepticism that the co‐primary requirement is necessary or practical, despite these endpoints not necessarily being met within the same patient.
2. It was reinforced that while gluten exposure is problematic, symptoms and intestinal damage induced by short‐term gluten challenges during clinical trials will resolve when a gluten‐free diet is resumed. The Hawthorne effect complicates trial design and may require low dose gluten exposure during the study period to interpret primary endpoints, especially histology.
3. While a trial length of 52 weeks is recommended to assess the durability of drug efficacy and safety, the draft FDA guidance [4] allows for primary endpoints to be assessed after 24 weeks. Further discussion is needed to better understand if studies of this duration are necessary when FDA guidance for other symptom‐directed indications such as irritable bowel syndrome allows primary endpoints supported by substantially shorter duration trials and does not require a follow‐on 6‐month period [22].
4. If a drug demonstrates effect on both co‐primary endpoints in a symptomatic population and receives approval as an adjunctive treatment for coeliac disease, the information on the histologic co‐primary endpoint would be relevant for treating asymptomatic patients who have active histologic findings.

At the end of the meeting, there was interest in scheduling a future meeting to specifically discuss drug design for the paediatric coeliac population and to consider biomarker development. Specifically, it will be important to consider age‐appropriate endpoints, drug dose selection and safety considerations that may be specific to growth and development. It will also be important to discuss potential unique challenges of clinical trial design for children.

**TABLE 1:** Key considerations and unresolved questions arising from the Beyond Celiac Coalition Stakeholder meeting (May 2024).

Theme	Key considerations	Unresolved questions
Co‐primary endpoints	• FDA representatives maintained that, to support efficacy, a successful trial must achieve statistical significance for both primary endpoints, patient reported outcomes and quantitative histology, to support a therapeutic label claim in coeliac disease.	• For others in the Coalition, there was continuing scepticism that the co‐primary requirement is necessary or practical, despite these endpoints not necessarily being met within the same patient.
Gluten challenge and the Hawthorne effect	• While gluten exposure is problematic, symptoms and intestinal damage induced by short‐term gluten challenges during clinical trials will resolve when a gluten‐free diet is resumed.	• The Hawthorne effect complicates trial design and may require low dose gluten exposure during the study period to interpret primary endpoints, especially histology.
Trial length	• A trial length of 52 weeks is recommended to assess the durability of drug efficacy and safety; the draft FDA guidance [4] allows for primary endpoints to be assessed after 24 weeks.	• Further discussion is needed to better understand if studies of this duration are necessary when FDA guidance for other symptom‐directed indications such as irritable bowel syndrome allow primary endpoints supported by substantially shorter duration trials and do not require a follow‐on 6‐month period.
Extrapolation to asymptomatic patients	• If a drug demonstrates effect on both co‐primary endpoints in a symptomatic population and receives approval as an adjunctive treatment for coeliac disease, the information on the histologic co‐primary endpoint would be relevant for treating asymptomatic patients who have active histologic findings.	• Not applicable.
Future considerations	• Interest in scheduling a future meeting to specifically discuss drug design for the paediatric coeliac population and to consider biomarker development.	• Age‐appropriate endpoints, drug dose selection and safety considerations that may be specific to growth and development; potential unique challenges of clinical trial design for children.

*Note:* Points reflect discussion, not formal consensus or the official positions or policies of the FDA.

Limitations include (1) this meeting was an exploratory stakeholder meeting rather than a consensus‐building meeting; (2) potential bias in participant selection; (3) quantitative data were not generated.

## Author Contributions

**Robert Anderson:** conceptualization, writing – original draft, writing – review and editing. **Nelson Cheinquer:** conceptualization, writing – review and editing. **Kate Avery:** conceptualization, writing – review and editing. **Marie E. Robert:** conceptualization, writing – original draft, writing – review and editing. **Edwin Liu:** conceptualization, writing – original draft, writing – review and editing. **Joseph A. Murray:** conceptualization, writing – original draft, writing – review and editing. **Kristie Grebe:** conceptualization, writing – review and editing. **Jocelyn Anne Silvester:** conceptualization, writing – original draft, writing – review and editing. **Lisa M. Fahey:** conceptualization, investigation, writing – review and editing, writing – original draft, methodology. **Daniel A. Leffler:** conceptualization, writing – review and editing. **Bhargav Tulabandula:** conceptualization, writing – review and editing. **Beyond Celiac Coalition:** conceptualization, writing – original draft, writing – review and editing, methodology, supervision.

## Funding

The authors have nothing to report.

## Disclosure

Lisa M. Fahey: None. Robert Anderson: Shareholder and Director, Novoviah Pharmaceuticals Pty. Ltd., Brisbane Queensland Australia. No grants; Shareholder and Director, JM Clinical Pty Ltd, Brisbane, Queensland Australia. No grants; Consultant to: Topas Therapeutics GmbH, Hamburg, Germany; Takeda Pharmaceutical Company Ltd/Millennium Pharmaceuticals, Cambridge MA USA; Forte Bioscience Inc., Dallas TX, USA; Barinthus Biotherapeutics, Germantown, MD, USA; Immunic AG, Gräfelfing, Germany; Universal Cells—ASTELLAS LLC US, Seattle, WA, USA; AMYRA Biotech AG, Basel, Switzerland; DBV, Paris, France; EVOQ Therapeutics, Ann Arbor, MI, USA; Kanyos Bio Inc., Cambridge, MA, USA; Kerna Ventures; Catalys Pacific; Georgiamune Inc.; Sonoma Biotherapeutics Inc., South San Francisco, CA, USA; Switchback Therapeutics Inc.; TregTherapeutics Inc., NC, USA. Edwin Liu: Takeda consultant, UpToDate contributor. Joseph A. Murray: None. Marie E. Robert: Consultant for Takeda, Anokion, Alimentiv, Teva, BMS. Salary support from AstraZeneca. Jocelyn Anne Silvester: Consulting (last 5 years): Alimentiv, Chugai Pharmaceuticals, GLG, Guidepoint, Lumanity, Takeda Pharmaceuticals, Teva Pharmaceuticals. Research Grants to Institution (last 5 years): Amgen, Glutenostics LLC, Janssen, Milky Way Life Sciences, Takeda Pharmaceuticals. Foundation Grants (last 5 years): Allan Foundation, American College of Gastroenterology, Beyond Celiac, Celiac Disease Foundation, Chan Zuckerburg Initiative, Global Autoimmune Institute, North American Society for Pediatric Gastroenterology, Hepatology and Nutrition. Kate Avery: Consultant to Chugai Pharmaceuticals. Nelson Cheinquer: Employee of Takeda Development Center Americas and is a stockholder in Takeda Pharmaceutical Company Limited. Kristie Grebe: At time of the meeting, was an Anokion employee and shareholder, currently Navigator Medicines employee and shareholder. Bhargav Tulabandula: Employee of Takeda Development Center Americas and is a stockholder in Takeda Pharmaceutical Company Limited. Daniel A. Leffler: Employee of Chugai Pharma; Board Member of Foundation for Celiac Disease Outcome Measures.

## Conflicts of Interest

Potential conflicts of interest are outlined above in the disclosure section.

## Data Availability

Data sharing was not applicable to this article as no datasets were generated or analysed during this study.
